# Gaps in the congenital syphilis prevention cascade: qualitative findings from Kern County, California

**DOI:** 10.1186/s12879-022-07100-3

**Published:** 2022-02-05

**Authors:** Eunhee Park, Julie Yip, Emily Harville, Marlene Nelson, Gloria Giarratano, Pierre Buekens, Jennifer Wagman

**Affiliations:** 1 Department of Community Health Sciences, UCLA Fielding School of Public Health, 650 Charles E. Young Drive South, Los Angeles, CA 90095 USA; 2 Department of Bioengineering, UCSD Jacobs School of Engineering, 9500 Gilman Drive, La Jolla, CA 92093 USA; 3 Department of Epidemiology, Tulane School of Public Health and Tropical Medicine, 1440 Canal St. #8318, New Orleans, LA 70112 USA; 4 Partners in Care Foundation, 5251 Office Park Dr, Bakersfield, CA 93309 USA; 5 School of Nursing, Louisiana State University Health Sciences Center, New Orleans, LA 70112 USA

**Keywords:** Congenital syphilis, Syphilis screening, Sexually transmitted infection, Prenatal care, Social determinants of health, Qualitative methods

## Abstract

**Background:**

Congenital syphilis is preventable through timely access to prenatal care, syphilis screening and treatment of pregnant women diagnosed as infected. In 2018, California had the second highest number of congenital syphilis cases in the United States (U.S.), a nearly twofold increase in cases since 2014. This study assessed gaps in preventing congenital syphilis in the high morbidity region of Kern County, California.

**Methods:**

Between May 2018 and January 2019, we conducted five focus group discussions with pregnant/postpartum women and ten semi-structured interviews with prenatal care providers in Kern County. Focus group and interview data were recorded, transcribed, and analyzed to identify emergent themes pertaining to facilitators and barriers at each step (prenatal care, syphilis screening and treatment) in the congenital syphilis prevention cascade.

**Results:**

Gaps in congenital syphilis prevention discussed in focus group discussions with pregnant/postpartum women were related to limited prenatal care access, social-, economic-, and cultural-barriers, and substance use and co-occurring intimate partner/domestic violence. The gaps identified from interviews with prenatal care providers included social economic vulnerabilities of pregnant women and stigma and shame around the vulnerabilities, distrust in medical system, prenatal substance use, limited prenatal substance use disorder treatment facilities, and inadequate provider training on context-specific congenital syphilis management strategies. Gaps in partner notification, screening and treatment for syphilis were brought up by pregnant/postpartum women and prenatal care providers.

**Conclusions:**

Congenital syphilis continues to increase in Kern County and throughout the U.S. In high syphilis morbidity areas, comprehensive and tailored public health approaches addressing setting-specific gaps in prenatal screening and treatment are needed.

**Supplementary Information:**

The online version contains supplementary material available at 10.1186/s12879-022-07100-3.

## Introduction

Prevention of congenital syphilis (CS) is critical given that untreated syphilis during pregnancy may lead to miscarriage, still birth, or blindness, deafness, and/or bone deformities in the infant [1]. While CS can be fully prevented through timely syphilis screening and adequate treatment during prenatal care, the number of CS cases in the United States (U.S.) continues to rise [2]. There were 1306 CS cases reported in 2018, representing a 185% increase since 2014 [3]. The recent rise in reported CS cases is geographically concentrated as only 5% of U.S. counties had more than one reported CS case in 2018 [4, 5].

In 2018, California was the U.S. state with the second highest number of CS cases (332 cases), after Texas (367 cases) [3]. Of the 332 CS cases in California, the highest numbers were reported in Los Angeles County (64 cases), followed by Kern County which reported 56 cases. In comparison, Kern County had only 4 CS cases in 2013. The sharp rise of CS cases in Kern County paralleled an increase in early syphilis infection among women during the same time period [6]. The rate of early syphilis infection (i.e., primary, secondary, and early latent syphilis [7]) among women of reproductive age between 15 and 44 years old in Kern County tripled between 2013 and 2018 [8].

Kern County represents an important population for targeted CS prevention efforts in California. Although it is only home to 2.3% of the state’s entire 2018 population [9], 17% of all CS cases in California were reported in Kern County that year [6]. Kern County spans the southern end of the Central Valley of California. It has a diverse and growing population and the region is a top producer of agricultural goods and petroleum and the county’s economy is tied to both industries [10]. Slightly more than half (54%) of the Kern County population identifies as Hispanic and 45.3% of residents use a primary language other than English, most commonly being Spanish [11]. Twenty-one percent of the population lives below the poverty line (defined as an income below $13,064 for an individual under age 65) which is 1.5 times higher than the state average [11].

To assist in the implementation, monitoring and improvement of sexually transmitted infection (STI) treatment and prevention programs, cascade frameworks have been used to identify gaps in the steps required for effective testing and treatment (i.e., treatment cascade) and use of prevention methods (i.e., prevention cascade). Such frameworks are most commonly used in the HIV/AIDS field [12]. HIV treatment cascades are used to examine the sequential stages of care that persons living with HIV go through from initial HIV diagnosis to achieving viral suppression (i.e. diagnosed with HIV, receipt of HIV care, retained in HIV care, viral suppression) [13]. The model shows the proportion of individuals living with HIV who are engaged in care at each stage, allowing for identification of losses at or between different steps in the continuum, and for variations between different groups or locations. In 2015, the HIV treatment cascade was adapted to evaluate non-HIV STI programming efforts in Los Angeles County [14].

Similar to HIV treatment cascades, HIV prevention cascades are used to identify gaps in the steps required for effective use of prevention methods [12]. In 2018, Kidd et al. (2018) created a novel CS prevention cascade framework to analyze quantitative syphilis data an estimate the proportion of potential CS cases averted and develop a classification framework to understand gaps in prevention. Their study demonstrated that the largest CS prevention gaps existed (1) at entry to prenatal care and (2) between early testing and timely adequate treatment [15] and that “data on the underlying reasons for lack of early prenatal care are not available in the national case report surveillance data, and might vary by geographic area” (p.S28) calling for additional research to identify context-specific factors contributing to gaps in CS prevention [15].

The aim of current study was to engage with pregnant and postpartum women and prenatal care providers in Kern County, California to identify contextual factors contributing to the CS epidemic and explore gaps in access to and retention in prenatal care and syphilis testing and treatment in this region of Central Valley, California. We present findings from five focus group discussions with pregnant/postpartum women and ten interviews with prenatal care providers, conducted between May 2018 and January 2019. Data were analyzed utilizing an expanded version of the CS prevention cascade framework developed by Kidd et al. (2018), modified to interpret qualitative data. The results increase our understanding of context-specific factors contributing to the ongoing CS epidemic in Kern County, California. We highlight areas where efforts should be placed to strengthen weaknesses that exist at/between different stages in the cascade of prenatal syphilis screening and treatment.

## Materials and methods

Focus group discussions (Additional file 1) and in-depth interviews (Additional file 2) were conducted in Kern County, California and East Baton Rouge Parish, Louisiana as part of a two-site, qualitative study conducted collaboratively by researchers from the University of California San Diego (UCSD) School of Medicine, Tulane University School of Public Health and Tropical Medicine (Tulane), Louisiana State University, California State University Bakersfield, the March of Dimes and the U.S. Centers for Disease Control and Prevention (CDC). Due to unique distinctions in the CS epidemics, as well as gaps in CS prevention between the two sites, the results from East Baton Rouge Parish, Louisiana were published separately [16]. In this paper, we present findings from Kern County, California.

### Expanded congenital syphilis prevention cascade framework to identify linkage and retention gaps in high-risk pregnancy

**Fig. 1:**
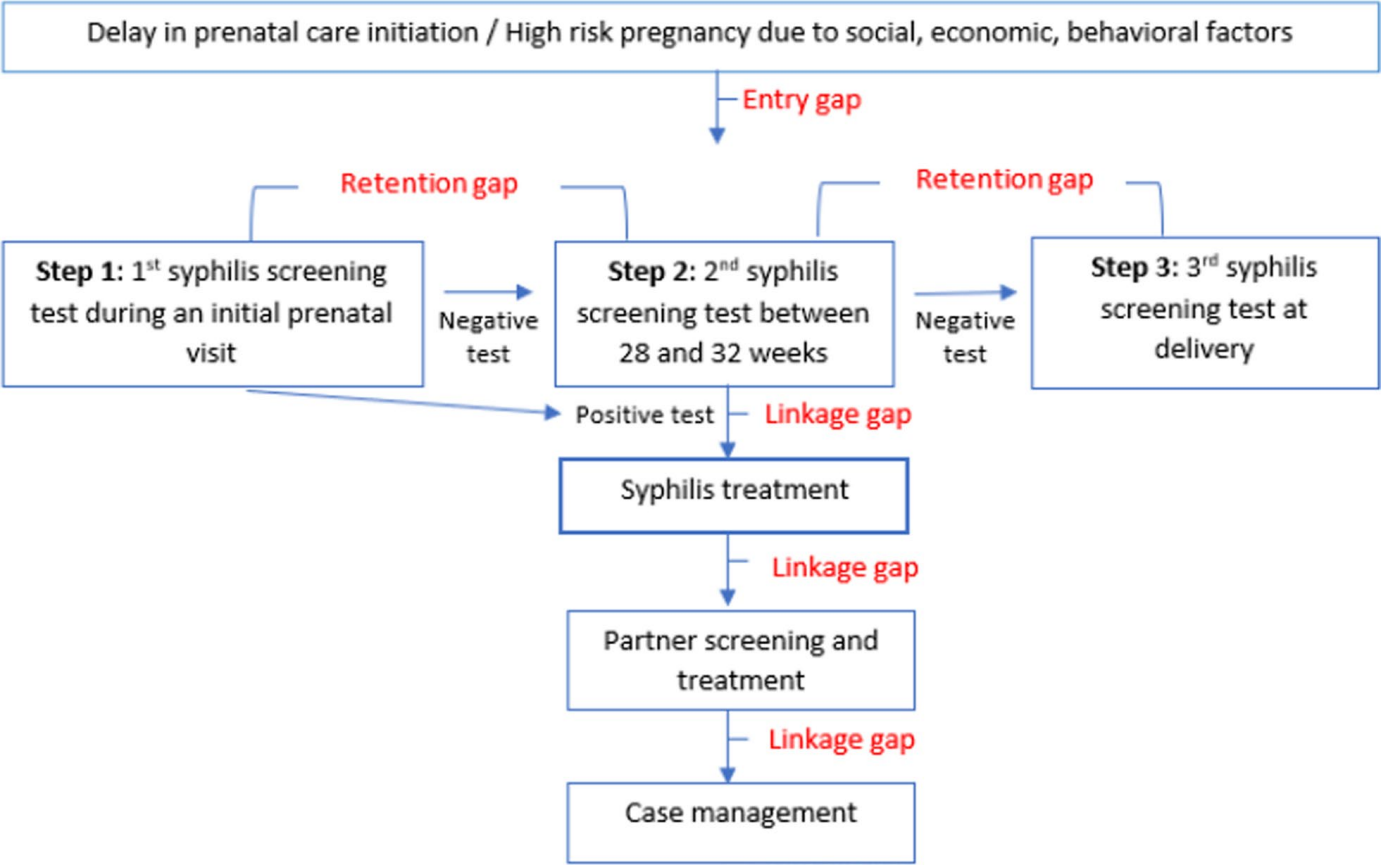
Congenital syphilis prevention cascade to identify entry, linkage, and retention gaps in high-risk pregnancy

Figure 1 illustrates the steps in the CS prevention cascade we adapted to organize our findings. This figure is referenced in an earlier publication, presenting results collected for this study in East Baton Rouge Parish, Louisiana [16]. Step 1 in the figure represents pregnant women being screened for syphilis during the first trimester. If infected with syphilis, they will be notified for their positive test result and receive treatment for syphilis. Women who are not infected with syphilis during the first trimester are advised to remain in prenatal care throughout their entire pregnancy. Step 2 indicates recommended re-testing for syphilis at 28–32 weeks’ gestation in areas of high CS morbidity like Kern County. If infected with syphilis, pregnant women should receive treatment for syphilis. Step 3 shows the third syphilis testing for a mother and infant after delivery. Partner notification is also highlighted as an essential component in the management and prevention of syphilis infection and refers to the practice of the newly diagnosed person notifying the sexual partner(s) that they may have been exposed to the infection. Case management refers to support programs for women with social or health concerns.

The first potential gap is referred to as an “entry gap.” It occurs if a pregnant woman presents late (i.e., after 32 weeks of gestation) for timely syphilis screening test or does not receive prenatal care at all until delivery. The next possible gap occurs between Steps 1 and syphilis treatment if syphilis is diagnosed but not treated. This is referred to as a “linkage gap” for a patient who failed to get timely, adequate treatment after the patient is notified with a positive syphilis test result. A possible “retention gap” occurs after a pregnant woman entered the prenatal care (between steps 1 and 2, and steps 2 and 3). If a pregnant woman doesn’t return for the next prenatal visit and/or syphilis screening, a retention gap occurs. Important additional gaps are possible if the pregnant patient is diagnosed with syphilis and does not notify her sexual partner(s), or a notified sexual partner(s) not getting screening or treated. Regardless of STI status, if a prenatal provider identifies other social, economic, behavior factors that could pose high-risk to pregnancy, the pregnant woman should be referred to proper case management providers.

### Focus group discussions with pregnant/postpartum women

Focus group participants were primarily recruited from five different locations/populations: (1) two major health care service agencies that provided pre- and post-natal health care (Omni Family Health and Clinica Sierra Vista); (2) a local residential substance use treatment program; (3) a transitional sober living community; and (4) an intimate partner violence (IPV) / domestic violence (DV) resource center. In each location/population, the study was introduced and presented in full detail by the research team to the manager or director of the agency/program/center. Some participants were recruited through snowball sampling, a nonrandom sampling technique whereby initial participants identify additional study participants. Brochures and information sheets containing full descriptions of the study, including contact information for the research team and details on how to get in touch for more information or to enroll in the study, were left with the manager/director. These materials were provided to staff at each center/location, who were asked to share details of the study with their clients/residents to solicit participation. Minimum eligibility criteria for focus group participants included: (1) Adult women (18 years and older); (2) receiving prenatal or early postnatal care in Kern County; (3) Having been a resident of Kern County for at least 6 months; (4) Currently pregnant or delivered an infant less than 12 months prior; (5) Having a phone or another way of being contacted; (6) Consenting to involvement in study; and (7) Being “high-risk” for CS which we defined as having any of the following characteristics: history of syphilis infection; history of incarceration; current or past drug use; multiple or concurrent sex partners in the past year. Focus groups were held at community-based locations (including the facilities from which participants were drawn) and public libraries. A total of 42 pregnant and postpartum women participated in the focus group discussions.

### In-depth interviews with prenatal care providers

A list of prenatal care providers was compiled from directories provided by the Kern County Department of Public Health, Omni Family Health, Clinica Sierra Vista, and Kern Medical Center. We invited providers to participate by sending emails to or telephoning them directly to inform them about the study and screen for eligibility. Eligibility criteria for in-depth interview participation were: (1) Prenatal care provider who worked in Kern County in their current position for more than 6 months; (2) Identified as possessing knowledge about the healthcare setting and dynamics relevant to CS and pregnant women in the region; (3) Currently working directly (at least 50% of the time) with high-risk pregnant women; (4) Having a phone or another way of being contacted; and (5) Consenting to involvement in the study. A total of 10 prenatal care providers meeting all eligibility criteria participated in interviews that were conducted in a private location or on the telephone.

All recruitment and data collection activities with pregnant/postpartum women and prenatal care providers were conducted between May 2018 and January 2019 by two bilingual (English–Spanish) master’s level researchers from California State University, Bakersfield and the Principal Investigator of the study who was a faculty member at UCSD. All participants were purposively recruited based on the eligibility criteria described above. As interested and eligible participants were contacted, a convenience sampling method was used to select those who responded first and participants were recruited until we reached data saturation (i.e., participants were no longer providing new information). To include perspectives of Spanish-speaking pregnant/postpartum women, since approximately half of the Kern County population identified as Hispanic with Spanish as their primary language [9], one focus group was conducted in Spanish. All focus group and interview data were gathered using semi-structured guides with open-ended questions to elicit information on knowledge and attitudes related to CS prevention and treatment; information-seeking behaviors; awareness of the high prevalence of syphilis and CS; forms of patient-provider communication; and challenges in accessing or providing prenatal care. All participants provided written informed consent and all data collection sessions were audio recorded with participants’ written consent.

In compensation for their time and transportation costs, pregnant/postpartum women received a $25 gift card and prenatal care providers received a $50 gift card. The research protocol and instruments were approved by the Institutional Review Boards (IRB) at UCSD and Tulane and final clearance was provided by the CDC’s Office of Management and Budget (OMB).

### Data analysis

Each audio recording of the focus group discussion and interview data collection sessions was transcribed verbatim into a Microsoft Word document. The focus group conducted in Spanish was first translated into English and later quality checked by a Spanish-speaking research assistant at UCSD (who was not involved with data collection) who made corrections as needed. All transcript files were imported into QSR NVivo V12. Thematic analysis was used to identify salient themes and develop codes [17]. Common themes that emerged from focus groups with pregnant/postpartum women were scheduling appointments, transportation barriers, time limitations and delays, social vulnerabilities (including poverty, homelessness, language barriers), substance use, relationship violence, fears of criminal justice, and lacking trust in social welfare system. Themes from prenatal care provider interviews included perceived barriers to pregnant women’s access to care, such as social economic vulnerabilities of pregnant women and stigma and shame around the vulnerabilities, distrust in medical system, prenatal substance use, limited prenatal substance use disorder treatment facilities, and inadequate provider training on CS. The data analysis team at UCSD read transcripts twice to generate initial themes and identify relevant codes which were refined continuously to reflect the data accurately. Approximately 25% of all transcripts were double coded by two separate researchers and compared to assess interrater reliability. Coding differences were discussed and resolved as a group.

## Results

Five focus group discussions were conducted with 42 pregnant/postpartum women. Fifty percent of pregnant/postpartum women were aged between 20 and 29 years, and 43% were between 30 and 39 years, with 40% identifying as Hispanic or Latina. More than 90% of pregnant/postpartum women had an annual income less than $15,000 (Table 1).

**Table 1:** Pregnant/postpartum women focus group participants (N = 42)

Category	*N*	%
Age		
20–29	21	(50.0)
30–39	18	(42.9)
40–49	3	(7.1)
Pregnancy status		
Currently pregnant	5	(11.9)
Pregnant past 12 months	37	(88.1)
Annual income		
Less than $15,000	39	(92.9)
Less than $20,000	1	(2.4)
$20,000 and more	1	(2.4)
N/A	1	(2.4)
Race/ethnicity		
Hispanic/Latina	17	(40.5)
White	15	(35.7)
Black/African American	5	(11.9)
American Indian/Alaska Native	3	(7.1)
Other	2	(4.8)
Focus group setting		
Rehabilitation centers	27	(64.3)
Hospital/Clinic	10	(23.8)
Community organizations	5	(11.9)
Focus group language used		
English	32	(76.2)
Spanish	10	(23.8)

Ten interviews were conducted with 4 male and 6 female providers, including obstetrician/gynecologists (Ob/Gyn), nurse practitioners, and maternal–fetal medicine specialists. All providers had been in their current (at the time of interview) position for at least 1 year. Four were deeply rooted in Kern county, reporting they had been in their current place of employment for more than a decade. Six providers were in a community or public clinic, 2 were in a single-specialty group practice and 2 were in a multi-specialty group practice (Table 2).

**Table 2:** Prenatal provider interview participants (N = 10)

Category	N
Age	
30–49	4
50–69	4
N/A	2
Gender	
Male	4
Female	6
Position	
Obstetrician/Gynecologist	2
Nurse/Nurse practitioner	5
Medical investigator	1
Health clinic administrator	2
Years in current position	
1–10	6
11–20	2
21 and more	2
Practice type	
Single-specialty group practice	2
Multi-specialty group practice	2
Community or public clinic	6

### Pregnant/postpartum women’s perspectives on initiating and staying in prenatal care and getting tested and treated for syphilis

The pregnant/postpartum study participants shared a broad range of perspectives surrounding their experiences with accessing and using prenatal care services throughout Kern County. All were aware of the importance of starting and staying in prenatal care throughout pregnancy. At the same time, however, they all talked about the numerous barriers they experienced in their lives, all of which were perceived to increase likelihood of falling into gaps along the cascade of CS prevention. We organize the findings in this section according to barriers reported, as they pertained to: (1) Prenatal care access, (2) Social, economic and cultural factors, and (3) Substance use and co-occurring intimate partner violence and domestic violence (IPV/DV).

#### Prenatal care access

Focus group participants consistently shared narratives about how pregnant women in Kern County struggle with getting timely and appropriate access to prenatal care services. Key barriers mentioned included long waiting times to schedule appointments and/or see a provider, health insurance limitations, and geographic and transportation-related obstacles.

*Long waiting times* Many women said gaps in their timely entry to and retention in prenatal care were caused by difficulty with scheduling appointments. Specifically, women were frequently told they would have to wait weeks or months to get in to see a provider and those who *did* finally go to a clinic usually faced long-wait times between their arrival and the start of their appointment.

*“I would say time frames. They wouldn't be flexible with the scheduling, so it would be, like, what they offered and they wouldn't really work with you on when you could go in.” – Participant from Focus Group #4*

*Health insurance limitation* Participants attributed long wait times and related retention gaps to not having health insurance or having health insurance with restrictions, including not being accepted by a particular provider who is located close to their home. Others felt there was a limited number of prenatal care providers available or willing to see them.

*“I didn’t get to actually see a doctor until two and a half months later [after I made an appointment call]. There were so many people [waiting to see the doctor] already. They are just backed up. Unless you have a private insurance, you’re not getting in any time soon.” – Participant from Focus Group #2*

*Geographic and transportation-related obstacles* Kern County is geographically spread out and many women felt healthcare facilities were too far from their houses and/or workplaces to travel back and forth for frequent visits. Long-distance travel was not possible given the many other responsibilities (e.g., work, taking care of children and family members) participants were balancing. Several additional transportation-related obstacles were narrated, including lack of a personal vehicle, what was perceived to be an inefficient public transportation system in Kern County, and costs of transportation. Although some health centers offered services like ridesharing and bus access, these options were not always possible or available to those in more rural areas of the region. All of these geographic and transportation-related obstacles contributed to both retention and linkage gaps.

*“Simply because of the transportation situation. You don’t make it to the appointments because it’s going to take too long on the bus or you won’t make it back in time to pick up the kids at school.” – Participant from Focus Group #3*

#### Social, economic and cultural factors

Although women in our study placed importance on protecting their own health, and the health of their children including those in utero, most said it was difficult to prioritize everyone’s well-being when simultaneously faced with more pressing, day-to-day social, economic, and cultural challenges.

*Unstable housing and homelessness* Many participants said they were marginally housed or homeless during their pregnancy and described how this instability made attending prenatal care appointments difficult, particularly if they had to go to different locations for syphilis testing and results notification. Lacking constant housing also made it hard to maintain stable relationships, contributing to gaps in partner communication and notification.

*“I was homeless, and when I found out I was pregnant, I didn’t go in right away to go see an Ob/Gyn. It actually happened, like, when I was 5 months…I didn’t do very well with going to appointments… I missed my appointments half the time.” – Participant from Focus Group #2*

*Economic instability* Unemployment and having a low-income were also both presented by participants as obstacles to accessing and remaining in prenatal care. Both of these economic factors were associated with barriers mentioned previously (including lack of health insurance, inability to pay for transportation to clinics, medical fees) and linked with retention and linkage gaps.

*Cultural barriers* Language was a main component of culture and with the diversity of Kern County, language-related barriers, including limited literacy obstructed some participants’ communication with providers. These obstacles were described in relation to gaps in all stages of pregnant women’s access to and use of prenatal care experiences. Some of the Spanish-speaking participants, for instance, struggled to understand key components of the health advice they received from medical professionals.

*“I*t was difficult because of the language. Almost the majority of pediatricians that I looked for do not speak my language. *It was difficult to be able to understand the doctor, or with my baby's pediatrician.*” – Participant from Focus Group #5 Conducted in Spanish

Others failed to complete welfare service applications and critical medical documents.

*“When they [pregnant women]’ve got to fill out a [welfare service] application, they can’t even read and write in Spanish. A lot of them. And it’s embarrassing and frustrating.” – Participant from Focus Group #3*

One woman reflected on how her own use of health services was negatively impacted by limited cultural competence among staff at the Department of Human Services. She felt they lacked training on how to interact effectively with people from diverse backgrounds to deliver services that meet the social, cultural and linguistic needs of patients, including those with limited reading and writing skills, both in English and Spanish.

*“They’ll give you information but it’s in English. How are you going to understand it? And you ask them “Is there a translation?” “No, we only have the information in English.” It's embarrassing for people and it's frustrating, especially when you've got somebody like that being rude.”* – Participant from Focus Group #5 Conducted in Spanish

#### Substance use and co-occurring intimate partner/domestic violence

Participants shared that a small but concerning number of women they knew used substances during pregnancy. Use of alcohol and marijuana were mentioned in focus groups and participants believed methamphetamines were the most commonly used illicit drug in Kern County, given their high availability and relatively low cost. Participants also felt methamphetamine and other drug use were germane to conversations about CS because it was believed to be associated with risky sexual behaviors like unprotected sex, and multiple-, concurrent sexual partners, and correlated to increase in vulnerability for STIs including syphilis.

*“A lot of people are shooting meth and going around and screwing everybody. If you are in a circle of people who uses drugs and have a very promiscuous lifestyle, being on drugs clouds your judgment. Just because you take care of yourself doesn't mean everybody else does.” – Participant from Focus Group #1*

Drug use was also perceived to be linked with mistrust in the health, criminal justice and social welfare systems, and experiences of IPV/DV. Participants said pregnant women using drugs were consistently afraid of being reported to or interacting with health providers who could report their drug use, police officers who could arrest pregnant women, and Child Protected Services (CPS) who could take their children away. These fears were all described as barriers to entering/remaining in prenatal care and significant obstacles to having any tests done that required collection of biological samples (e.g., blood, urine). Participants said pregnant women using drugs would commonly isolate themselves from others and skip prenatal appointments to avoid the risk of being detected, arrested, imprisoned or of losing their baby as a result of their substance use. Participants narrated how friends or relatives who used drugs during pregnancy had intentionally avoided prenatal care, leading some to give birth to babies with adverse health outcomes.

*“My friend and two other girls, their babies were born with syphilis. My friend was drinking a lot of alcohol so she didn’t go to the doctor. They all thought they were going to get in trouble. They thought they would be reported. Due to that, there are 4 babies that are…going to be mentally disabled in their life because their moms were too scared to go to the doctor because they were on drugs or they had warrants out for their arrest.” – Participant from Focus Group #2*

One participant narrated her own use of drugs during prior pregnancies and how it influenced her behavior during the recent pregnancy. She said she had become “paranoid” about going to prenatal care visits and having the requisite “pregnancy tests” completed as they posed the risk of her getting caught for drug use.

*“I used drugs throughout a lot of my pregnancies…I was PARANOID about like giving up my current pee because of the drugs in it. So I was giving him [doctor] old pee [during] the entire pregnancy…It's not hard to get prenatal care at all but the reason that a lot of us don't do the prenatal care in the beginning is because we're using drugs. I've done it with two of my kids. I didn't get prenatal care with her until I was like seven months and then with my other one I had no prenatal care but, I had prenatal care with all my other ones. I would also falsify on SOME of the [urine] tests.” – Participant from Focus Group #3*

Participants agreed that even when pregnant women who were using drugs were concerned with protecting their health through prenatal care, they were significantly more worried about the urine tests done during prenatal visits that could notify the provider of their drug use. Thus, this presented an obstacle to critical linkage steps in the CS prevention cascade.

Intimate partner violence and domestic violence (IPV/DV) was brought up in discussions as a perceived risk factor among women of reproductive age for both acquiring syphilis during pregnancy and transmitting it to their baby. Conversations on the links between STIs and IPV/DV, however, most heavily focused on increased vulnerability among women who used substances during pregnancy, particularly among young women who are under the age of 25 years. Participants believed women using drugs who had violent partners were afraid to seek “medical attention” for a few reasons. One, health providers are mandated reporters of IPV/DV and participants felt most abused women did not want their partner to be punished/arrested or have their children removed from the household.

*“I've heard on so many occasions where girls that are pregnant and they are afraid. They're afraid to go and seek medical attention because they are on drugs or there might be abuse going on. They're scared because they [providers] are mandated reporters. I know that there are a lot, a lot of girls that just strictly because they are afraid to have their child taken from them or to face the reality of the situation. [Women are] afraid to go to the doctor.*” *– Participant from Focus Group #4*

Second, abusive men were said to commonly control their pregnant female partner’s movement and this often included restricting them from going to appointments, including prenatal care visits. Third, women experiencing partner violence, whether in or out of the context of drug use, were said to be less likely to notify an abusive partner of potential syphilis exposure, out of fear of being blamed, hurt or punished in another way.

Participants spoke freely about how punitive approaches to substance use and IPV/DV deterred many pregnant women who used drugs from seeking all levels of prenatal care, as well as substance use treatment services. Explicitly addressing women’s concern regarding their drug use, treatment options, and scope of confidentiality was recommended by participants as a way to mitigate the pervasive fears that block many pregnant women who are using substances from accessing social, medical and prenatal care services.

*“If there was something in forms, in line, saying, "Hey, you're really not going to jail. We're trying to help you." The drug addicts out there who are pregnant or might be pregnant or have kids would be more open to going out there and getting HELP, instead of being scared to go get help for your child, and get it for you too.” – Participant from Focus Group #2*

### Providers’ perspectives on prenatal care and timely testing and treatment for syphilis

Each prenatal care provider interviewed was aware of the exponential increase in CS cases over the past few years in Kern County. It was their shared opinion that numerous factors contributed to the ongoing, uncontrolled epidemic. We organize findings from these interviews in three categories according to: (1) Providers’ perceptions of barriers complicating pregnant women’s ability to access syphilis screening and treatment; and (2) Inadequate prenatal care provider training on how to manage CS.

#### Providers’ perceptions of barriers complicating pregnant women’s ability to access syphilis screening and treatment

Providers identified a range of factors that reduced their patients’ ability to access, remain in and complete all recommended components of prenatal care, including all steps in the CS prevention cascade.

*Housing instability and economic vulnerabilities* Housing instability including homelessness was identified as a common challenge faced by pregnant women at high risk for CS. Providers narrated how their patients experiencing homelessness rarely received any prenatal care until the time of delivery and those who *did* come for prenatal care were hard to re-contact to arrange follow-up appointments. A common theme in interviews was a perceived urgency to focus on understanding how reproductive health services including prenatal care could be improved in a place like Kern County. Providers were concerned about their limited ability to access hard to reach, underserved pregnant women in the population who were geographically, culturally, and economically marginalized.

*“She was a homeless person in her 20's. She turned out to be having syphilis and gonorrhea. The test came back 2 days later after visit, and by then she was nowhere to be found. She didn't respond. It happens often.” – Interview #1, Ob/Gyn*

*Stigma and shame surrounding social vulnerabilities* Many providers observed how pregnant women using public assistance programs were more likely to experience gaps in the cascade of CS prevention. This was attributed to the geographic, socioeconomic and cultural factors mentioned from focus group findings. Additionally, providers believed lack of engagement in care was influenced by the strong and negative stigma surrounding the receipt of social welfare services, causing pregnant women to feel uncomfortable and embarrassed about seeking care from prenatal providers and revealing their use of social welfare benefits. They explained how issues surrounding their patients’ financial difficulties, as well as substance abuse often emerged during office visits and many pregnant women demonstrated feelings of shame surrounding these vulnerabilities.

*“They were couch surfing. Some of them were at the homeless shelters. Working with homeless women or IV-using moms can be delicate because there’s so much shame around it, because you’ll see these moms wait until they’re 40 weeks to go in with zero prenatal care.” – Interview #5, Medical investigator*

*Distrust in medical system* Another concern voiced by providers was that some pregnant women diagnosed with syphilis refused treatment, despite providers’ explanation about potential harms of untreated syphilis to a fetus.

*“We follow them if they come in at the first visit, … then we let them know we're still going to test you again in the second trimester or third trimester. This is the recommendations are not laws. We worked really hard on getting everyone on the same page including the women that are being tested… There are pregnant females that refuse to have treatment.” – Interview #4, Public health nurse*

Providers suggested that reasons for avoiding syphilis treatment be investigated in future studies, both among both pregnant women and their male partners who were also reported to have refused testing and treatment. One provider narrated that there is a big linkage gap between positive syphilis test results and follow-up treatments due to miscommunication between medical providers and pregnant women arising from some pregnant women’s substance use and distrust in medical system.

*Interviewer: After their last one [syphilis screening test], do they come back for a follow up for that [treatment, if tested positive for syphilis]?*

*Answer: That's a BIG opportunity for improvement I think. Because the patients that we have that have syphilis, they're not very compliant and they're not willing to WAIT in an E.D. for treatment. And I think that's a BIG issue. We always tell them to follow up with the Health Department and...if they need to come back here for treatment per recommendation from the Health Department, then of course we would treat them again. But sometimes the patients have psychiatric issues or have a problem with drug use. So they're not thinking or acting very normally. They'll come back and sometimes they can't even...communicate to what they're there for. So the communication--there's a lot of miscommunication sometimes and opportunities are missed--. – Interview #8, Ob/Gyn*

*Substance use* Similar to findings that emerged in the focus group discussions with pregnant/postpartum women, drug use was consistently brought up in the provider interviews as an issue intertwined with, and often worsening, other risk factors for CS, like transactional sex, incarceration, and poverty.

*“Actual true sex workers...the reality is that a lot of them are pregnant and stay from place to place for drugs. It is NOT sex work in a traditional definition… They're people that have a DRUG problem, and they're people that their bodies become a commodity. The exchange for money or drugs or a place to stay. Those are the hardest ones to track because when you go from spot to spot, staying with this person because they have what you need and then you get kicked out, you know, or that person gets arrested or whatever you go to the next place.” – Interview #5, Medical investigator*

Polydrug use (i.e., use of multiple types of illicit substances at one time) among pregnant patients was observed by numerous prenatal care providers who confirmed that methamphetamines were commonly used, in addition to opiates, alcohol, cigarettes, and marijuana.

*“I would say there are co-occurring, multiple substances. If they weren’t opiates, they may have been smoking weed. Or methamphetamines. We can point to that mothers drop off from smoking and other substances. But there are folks continue to use that. What we've seen or heard about a lot is alcohol and meth.” – Interview #10, Public health nurse*

Provider perspectives corroborated findings from focus groups, for instance that pregnant women using substances commonly avoided prenatal care visits to avoid tests that could reveal their drug use. Providers explained how difficult it was for them to ensure their patients received timely treatment and comprehensive prenatal care when their patients were facing such a large set of obstacles. They consistently referenced how the most at-risk pregnant patients in Kern County were lost at all stages in the cascade of CS prevention, beginning with those who did not appear for the first trimester visit, to those who were lost to follow-up and never presented for the third trimester testing.

*Limited substance use disorder treatment facilities for pregnant women* Protocols for managing pregnant women who tested positive for drug use, including weekly drug tests, supportive services to deal with withdrawal, helping patients adopt healthy life style choices through coaching and education were referenced during interviews. Several clinics in the region had drug treatment programs for pregnant women, including residential treatment services, but there weren’t enough resources to accommodate everyone in need.

*“The pregnancy protocol [for substance misuse] would be that...they are meeting with our in-house physician once a month for our program... they're on the treatment program and we would drug test them once a week until they gave birth.. They were being managed, and then they would we would be case-conferencing them, having an interdisciplinary team talking about their cases. Many people wanting to get into treatment, but not enough rooms... That's one thing that we can—we can make happen for them.” – Interview #8, Ob/Gyn*

While pregnancy could be leveraged as an opportunity to access drug treatment, one provider pointed out that few drug treatment facilities exist in the region, which they felt resulted in high levels of continued substance misuse among pregnant women and related maternal and congenital complications, with involvement of CPS as a last repercussion.

#### Inadequate prenatal care provider training on how to manage congenital syphilis

Although prenatal care providers said they received updated STI information from online guidelines provided by the CDC and the American College of Obstetricians and Gynecologists (ACOG), most felt their knowledge of how to effectively manage CS was inadequate. Almost all providers interviewed said they had not received formal training on CS since their medical residency, which for some was decades ago. This gap was felt to negatively impact providers’ ability to adequately mitigate the CS epidemic in the region and among their patients.

Given the critical importance of CS prevention, prenatal care providers expressed desire to receive focused, ongoing training on syphilis treatment guidelines for pregnant women, encompassing screening, testing, test interpretation, patient and partner notification, and the recommended follow-up treatment regimen in the clinic in which they practice.

*“I wish I had more I have been sent to a class or sent to a conference. I think it [syphilis screening test and treatment] could have been briefed up for me.” – Interview #2, Nurse practitioner*

*“I read CDC STD guidelines. I'm going according to that, but I have no formal training [focused on syphilis-specific training after residency.” – Interview #8, Ob/Gyn*

### Gaps in partner notification, screening and treatment for syphilis

Persons diagnosed with an STI are encouraged to notify their sex partner(s) to tell them about potential exposure and refer them for evaluation and treatment. Nonetheless, obstacles to communicating this information were repeatedly brought up by both pregnant/postpartum women and prenatal care providers as a gap in the CS prevention cascade in Kern County (Fig. 1). Promotion of conversation about and testing for syphilis were felt to be difficult due to intense stigma surrounding STIs. Common words women used when talking about STIs were “dirty” and “unclean” and providers emphasized how extensive moral judgement was placed on women and men who acquire an STI.

Most pregnant and postpartum women suggested they made efforts to notify partners but shared how difficult it was to persuade their husband/sex partner to get screened for syphilis, for a variety of reasons, including stress and stigma surrounding STIs and testing.

*“It took me a lot to convince my husband…I told him “I want you to go and get an exam” [He said] “But, why? I am healthy.” I told him “Because it is important.” He said “You can do it, but I am good. Mistrusting my husband and everything until after the test result was revealed. His test took about two weeks for it to come, and those two weeks were eternal.*” – Participant from Focus Group #5 Conducted in Spanish

Prenatal care providers also suggested they took steps to encourage their pregnant patients to notify their sex partner(s) about potential syphilis exposure and urge them to undergo testing and treatment. However, it was common perception that partner tracing, testing and prevention education were the responsibility of the local health department. Providers believed public health investigators were better placed to communicate with and influence the behaviors of their pregnant patients’ sex partners, and that prenatal care providers had obstructed ability to be effective in this step of the prevention cascade. Providers often felt the most they could do was encourage their patients to tell their sexual partners and suggest they get tested.

*I don't know what the Public Health law is in that regard. I do know that people will refuse the treatment. I've heard in one of our meetings, where a woman was tested positive and acknowledged that her partner was positive. They both knew it, but they didn't want to be involved in any long-term engagement with the provider. They just wanted to get back to their lives that they would deal with it themselves. Time and time again, they were well aware of it but they chose to take that risk.” – Interview #6, Public health nurse*

## Discussion

This study explored gaps in access to and retention in prenatal care and syphilis testing and treatment in Kern County, California where, in 2018, the rate of new CS infections was the second highest in the state and 494% higher than the state average [6]. Our findings demonstrate the utility of applying an adapted version of the CS prevention cascade framework [15], modified for use in qualitative research. We were not the first to adapt this model in such fashion. Investigators in Louisiana previously modified it for their own qualitative assessment of barriers and the public health response to CS in Caddo Parish [18]. While we did *not* use Kidd and co-authors’ framework to estimate the proportion of potential CS cases averted, the design of our analysis plan was based on their classification model to identify gaps in CS prevention [15] allowing us to identify where improvements are needed and where CS prevention efforts should be prioritized in Kern County.

Our results contextualized national findings indicating the largest gaps in CS prevention in the U.S. were at entry into early prenatal care and between early testing and timely, adequate treatment [15]. Similar findings emerged in California, through surveillance data from 2012–2014 suggesting CS case mothers were more likely to initiate prenatal care in the third trimester, relative to non-CS case mothers [19]. Through our focus groups with pregnant and postpartum women and interviews with prenatal care providers, we identified three main domains from which high-risk pregnant women’s late entry into prenatal care were associated. *First, access to care* was a substantial challenge for underserved pregnant women who reported they commonly experienced long wait times to make appointments and during in-clinic visits, geographic and transportation-related barriers and health insurance limitations. Most focus group participants were either uninsured or covered by government-supported health care with many restrictions. Similar findings have emerged in two sites in Louisiana—Caddo Parish [18] and East Baton Rouge Parish [16] where delays in care for pregnant women were associated with health care insurance coverage, and women’s struggles to find providers who accepted patients with low-cost, subsidized or no insurance. *Second, a clustering of social, economic and cultural barriers* challenged women’s entry and retention in care, ranging from unemployment and living with homelessness, to unmet linguistic needs. Research with CS case mothers in Indiana also found housing instability interfered with recommended care during pregnancy [20]. Access to culturally competent prenatal care seemed unjustly limited for many women in our study. Several participants felt their interactions with health providers while pregnant were undignified and the environments at clinics were negative and non-supportive, detrimentally impacting women’s ability to achieve health goals and often discouraging continued use of services. *Third, substance use and intimate partner / domestic violence* contributed to gaps in all steps of the CS prevention cascade.

Findings from the current study also expanded understanding of factors contributing to gaps between early testing and timely, adequate treatment for syphilis. Most participants were aware that some pregnant women who undergo syphilis testing and get a positive diagnosis do not receive adequate treatment before delivering their baby. National data suggested this gap often results from failure to initiate timely syphilis treatment [15]. Potential underlying causes of prenatal syphilis treatment gap were found—in our research—to be the same as those that obstructed pregnant women from engaging in prenatal care in the first place. Reasons included the time and cost of transport to a clinic, waiting times in clinics, housing instability, financial struggles and unemployment, other competing life priorities, cultural barriers, and substance use and co-occurring IPV/DV. All of these barriers were exacerbated if testing and treatment could not be done at the same time or in the same location. Additionally, it is possible some women were lost between testing and treatment as a result of prenatal care providers lacking post-medical school STI training and/or having limited, or no, experience with diagnosing and treating syphilis. The CDC recommends all prenatal care providers be trained to complete a sexual history for their patients, test all pregnant women for syphilis, treat women with syphilis immediately, confirm syphilis testing at delivery and report all cases of syphilis and CS to the local or state health department [7].

Another explanation offered for increased rates of CS in the U.S. was that women who test negative for syphilis infection or test positive and receive adequate treatment early in pregnancy can acquire new syphilitic infections later in gestation [15]. In our study, both pregnant women and providers explained how there was very little follow-through to ensure male partners potentially exposed to syphilis were tested and linked to treatment. Thus, it is possible that women were re-infected during a later stage of pregnancy (i.e., through re-exposure to a partner who remains positive). Research from Louisiana identified a related challenge in that even when providers *did* notify male sex partners of potential syphilis exposure, they typically could not offer them treatment at the same prenatal clinic, precluding their ability to know if the partner was linked to an adequate regimen of treatment [7].

Historic and ongoing intersections between drug use and STIs in the U.S. are well documented. Before the pandemic, the CDC found the national prevalence of methamphetamine use in heterosexual women with syphilis increased from 6% (in 2013) to 17% (in 2017) [21]. Rates of primary and secondary syphilis among heterosexual women of reproductive age in the U.S. doubled during approximately the same time period (i.e., 2014–2018), and a directly correlated increase in new CS cases was concurrently observed [3]. Extensive research focused specifically on health outcomes during pregnancy in the U.S. found substance use was associated with mother to child transmission of syphilis [19, 22].

Our findings indicated many women dealing with substance use disorder (SUD) during pregnancy were also exposed to IPV/DV. The co-occurrence of violence, SUD, and STIs were well documented [23–26] and referred to as the sex, drugs, and violence (SDV) syndemic [26]. This reference drew on the syndemic theory which was proposed by Merrill Singer to account for co-occurring social and health issues that mutually enhance and exacerbate negative consequences of each condition [27–29]. While participants in the current study did not extensively discuss exposures to, observed accounts of, or experiences with handling the SDV syndemic, a complex relationship clearly existed between violence, drugs and reproductive health. Methamphetamine and alcohol use were most commonly mentioned in connection with pregnant women’s experiences of partner violence in Kern County. Further, participants felt IPV/DV and SUD were both a cause and consequence of syphilis. The physiological and behavioral effects of drug use were consistently linked with increased risk for abuse and injury, sexual risk taking (such as having unprotected intercourse), and transacting sex for money or drugs, all of which heighten vulnerability for STIs. Participants reported pregnant women with syphilis who used drugs and/or experienced IPV/DV were less likely to use prenatal services or undergo biological testing of any kind due to mistrust in providers, fears of having their drug use or exposure to IPV/DV identified and reported to authorities, and fears of having their children taken away by CPS.

## Conclusion

Strengths of the study include application of CS prevention cascade to qualitatively identify context-specific entry, linkage, and retention gaps in high CS morbidity area, incorporating perspectives from both pregnant/postpartum women and prenatal care providers, inclusion of Spanish-speaking pregnant/postpartum women, recruitment of vulnerable population (e.g. women with history of syphilis, history of incarceration, current or past drug use, multiple or concurrent sex partners), and utilization of systematic interview and focus group guides developed by multiple researchers from six research and public health institutions.

Our study has limitations that are important to note. First, this research is qualitative in nature and include only a small number of pregnant/postpartum women and prenatal providers. Thus, we are unable to comprehensively assess risk factors for CS or gaps in its prevention across Kern County. Further, given the magnitude of the CS epidemic in our study setting, our findings may not be generalizable to other counties or states characterized as lower morbidity regions. Our findings might also not apply to settings with different geographic, racial, and ethnic makeups. The second limitation is that not all of our focus group participants were CS case mothers. Thus, it is possible our findings overlook critical risk factors for CS, as well as gaps in the prevention cascade.

We suggest prenatal care providers be trained to identify at-risk women for experiences of IPV/DV and SUD by using evidence-based procedures for screening, following by provision of short-term mechanisms of support and proactive referral to comprehensive services for violence and substance use treatment. We advocate against punitive approaches with pregnant women who are using substances, as these have been associated with rising trends of CS in the region [30]. California is one of three states in the U.S. along with Hawaii and Washington State that does not consider fetal exposure to drugs as evidence of child abuse, but requires postnatal drug assessment of newborns. Babies with positive toxicology results for syphilis are referred to CPS [31] and stillborn cases associated with maternal substance use could lead women to be charged with child abuse, assault, manslaughter, and murder [32]. Instead of criminalization, research strongly suggests women-centered approaches are more beneficial for both the mother and the baby. Interventions for pregnant women with SUD can be strengthened by input from both prenatal care providers and those from more of the criminal justice/social welfare space (e.g., CPS case managers, prosecutors) so long as mothers are provided opportunity to demonstrate their parental capacity and interest [30]. Comprehensive case management services for social vulnerabilities and substance use have been found to be most beneficial when they are trauma-informed and women-centered (e.g., allowing children to accompany their mothers, involving peer support, providing women-only residential facilities) and we suggest prenatal health centers consider adopting these approaches as standard operating procedures [33–35].

Simplifying the steps in the CS prevention cascade for pregnant women at high risk for CS is also recommended. Research and experience have found that multiple visits and lengthy time required for syphilis testing and treatment contribute to gaps in reaching pregnant women infected with syphilis, as well as their sex partner(s). Immediate access to test results has also been suggested for decreasing loss to follow up [1, 19].

## Supplementary Information

**Additional file 1.** Screening form and guide: Pregnant Women.

**Additional file 2.** Screening form and guide: Prenatal Care Providers.

## Data Availability

The datasets generated and/or analyzed during the current study are not publicly available due to the risk of identification of participants, which may compromise the confidentiality of participant information, but are available from the corresponding author on reasonable request. An interview guide and focus group discussion guides are publicly available.

## Abbreviations

ACOG: American College of Obstetricians and Gynecologists
CDC: U.S. Centers for Disease Control and Prevention
CS: Congenital syphilis
CPS: Child protective services
IPV/DV: Intimate partner violence/domestic violence
Ob/Gyn: Obstetrician/gynecologists
STI: Sexually transmitted infection
SUD: Substance use disorder
