# Supplementary material for: Gaps in the congenital syphilis prevention cascade: qualitative findings from Kern County, California
Source: BMC Infect Dis. 2022 Feb 5;22:129. doi: 10.1186/s12879-022-07100-3 (PMC8818245; doi:10.1186/s12879-022-07100-3)
Supplement: Supplementary file 2 — Additional file 2. Screening form and guide: Prenatal Care Providers. [file 12879_2022_7100_MOESM2_ESM.docx]

**Screening Form: Prenatal Care Providers**

Hello, my name is _____________ and I am a research assistant. On behalf of the University of California / Tulane University, I am helping to implement a study with health care professionals who provide antenatal care to pregnant women. We aim to learn about current antenatal care practices in regions of the United States with high prevalence of sexually transmitted infections (STIs) like syphilis. Over the past 5 years there has been an increase in the rate of congenital syphilis cases in Kern County / East Baton Rouge Parish. The purpose of this study is to develop guidelines for health care users to effectively communicate with their patients at highest risk for syphilis infection/transmission so as to offer the best prevention and treatment services.

Some of the antenatal care professionals we speak with today will be invited to participate in a telephone or in-person interview that will pay an honorarium of $50, for about 60 minutes of your time. The interviews will be scheduled during the weeks of _____________ 2018. Are you interested in participating, and do you have some availability in that timeframe?

Yes 🡪 *Continue*

No 🡪 *Exit script*

May I ask you a few questions to see if you qualify?

1. Have you participated in any focus groups or been interviewed for any opinion-research studies about syphilis or congenital syphilis in the past 6 months?
   1. No 🡪 *Continue*
   2. Yes 🡪 *Exit script*
2. What is your current position?
   1. Obstetrician/gynecologist (OB/GYN)
   2. Family practice doctor (family physician)
   3. Maternal-fetal medicine (MFM) specialist
   4. Certified nurse-midwife (CNM)
   5. Family nurse practitioner (FNPs)
   6. Women's health nurse practitioner (WHNP)
3. On average, how many hours per week do you currently spend in direct antenatal patient care?
   1. 40 or more 🡪 *Continue*
   2. 20-39 🡪 *Continue*
   3. Less than 20 🡪 *exit script*
4. Which best describes the setting in which you practice? [Recruit a mix]
   1. Solo practice
   2. Single-specialty group practice
   3. Multi-specialty group practice
   4. HMO or Managed Care Organization (MCO)
   5. Ambulatory care center of hospital/medical center
   6. Community or public health clinic
   7. University or college student health facility
   8. Urgent care facility
   9. Government health facility (e.g., Veterans Administration)
   10. Other (please specify): ____________________________
5. How many years have you been in practice?
   1. More than 5 years - Recruit at least 4 per patient racial/ethnic segment 🡪 *Continue*
   2. 1 to 5 years - Recruit no more than 5 per patient racial/ethnic segment 🡪 *Continue*
   3. Less than 1 year 🡪 Thank and terminate
6. Have you worked in [Kern County/ East Baton Rouge Parish] for at least 12 months?
   1. Yes 🡪 *Continue*
   2. No 🡪 *Exit script*
7. We’re interested in talking to primary care professionals who serve patients from a range of racial and ethnic backgrounds. Approximately what percent of your patients are from the following groups (Prompt for each racial/ ethnic group below):
   1. White or Caucasian
   2. Black or African American
   3. Hispanic or Latino
   4. Native American
   5. Asian American

If patients are primarily Hispanic/Latino or Asian American:

What is the primary language most of your patients speak? ________________

1. Would you describe the majority of your patients as being in a:
   1. Lower socio-economic level
   2. Middle socio-economic level
   3. Higher socio-economic level
2. On average, how many female patients with a diagnosis of syphilis do you see each quarter (i.e., in a 3-month period)? This may be a diagnosis based on results from a blood test or from a test of fluid from a syphilis sore, or a combination of these.
   1. More than 10 patients 🡪Continue
   2. 5 to 10 🡪Continue
   3. Fewer than 5 🡪Continue
   4. None 🡪Thank and terminate;
   5. Don’t know 🡪Thank and terminate
3. Approximately how many of the patients that you see are uninsured or publicly insured (such as through Medicaid or the Affordable Care Act / ObamaCare)? *[Prompted]*
   1. A few
   2. Some
   3. About half
   4. Most
   5. All
   6. Don’t know
4. **Now I’d like to tell you the types of antenatal care providers we’re seeking for this study. Can you suggest any colleagues who might fit these criteria?** *If the respondent qualifies for the study, specify that referrals need to be someone outside their office or practice. If the respondent does not qualify, referrals to colleagues from the same office or practice are fine*.

Read criteria:

- 1. Obstetrician/gynecologist, family practice doctor, maternal-fetal medicine specialist, certified nurse-midwife, family nurse practitioner, women's health nurse practitioner
  2. Spends 20 hours or more each week in direct patient care
  3. Has been in practice at least one year, and ideally five years or more
  4. Has been working for at least one year in Kern County / East Baton Rouge Parish
  5. Sees at least one patient every 3 months with a syphilis diagnosis

Record contact information for any referrals.

***If eligible:***

Great, thanks for answering my questions! Based on what you told me, it looks like you are eligible to participate in our study! For next steps, we just need to schedule you for a phone / in-person interview. The interview will last about 60 minutes, and you will be compensated with a $50 gift card for your time. The interview will take place outside of your normal business hours, at a time convenient to you. Would you like to participate?

Yes 🡪 *Continue to schedule participant*

No 🡪 *Ok, thank you for your time.*

***If NOT eligible:***

Thank you so much for your time. Based on your responses, it appears that you are not eligible for the study we are conducting. Thank you for your time.

**Semi-structured Interview Guide for Prenatal Care Providers**

**Introductions:** Hello, my name is _______________________ and I work at [UNIVERSITY]. We’re partnering with the March of Dimes and CDC to learn how best to support the health and well-being of pregnant women in Kern County/ East Baton Rouge Parish. We want to understand more about your professional practices related to STD prevention and treatment, and where you access information about STD prevention. Please feel free to speak openly. Everything we talk about will be kept confidential. Let me know if you need to stop for any reason. If there are any questions you do not feel comfortable answering, that is okay; we can skip to the next question. You are also free to end this interview at any time. As a thank you for your time, we will send you a $50 gift card. We would like to record this interview so we can refer back to it later. All tapes will be destroyed after we have finished our analysis. Is it ok for me to record our conversation? [*If participant says no, thank him/her and end conversation].* Do you have any questions for me before we begin?

**Demographics**

**Indicate informant’s gender**

1. **Work Experience**
   1. Tell me about your experience providing prenatal care to pregnant women in Kern/ East Baton Rouge Parish.
      1. Where do you work?
      2. What are your main daily responsibilities?
      3. How long have you been providing prenatal care there?
   2. Approximately how many pregnant women do you work directly with each week?
   3. What percentage of your pregnant patients initiate prenatal care in the second trimester? Third trimester?
      1. What percentage of your pregnant patients frequently miss their appointments?
      2. What percentage are homeless or marginally housed?
      3. Do you care for pregnant patients who smoke, drink alcohol or use drugs? What kinds of drugs are the biggest problem among this population in your area?
      4. *If not mentioned…*Is alcohol a big problem among this population in your area? Smoking?

As I mentioned in the introduction, we are going to spend some time talking about professional practices related to STD prevention, including syphilis and congenital syphilis. As a reminder, all of the information we collect will be completely confidential and you can be totally honest in your responses. First we’re going to talk about any training or guidance you have received on testing and treatment for syphilis and congenital syphilis.

1. **Knowledge**
   1. Have you ever received any formal training or education about *testing* pregnant women for syphilis?
      1. If yes, What did the training consist of? *Probe – Classroom training, online certification, continuing education credits?*
      2. When did you receive your most recent training?
      3. Where did you receive the most recent training? Is this the same place you work now?
   2. Have you ever received any formal training or education about *treating* pregnant women for syphilis?
      1. If yes, what did the training consist of? *Probe – Classroom training, online certification, continuing education credits?*
      2. When did you receive your most recent training?
      3. Where did you receive the most recent training? Is this the same place you work now?
   3. Do you want more information or training related to diagnosis and treatment of syphilis?
      1. What types of information / training would be most helpful?
      2. Are there any topics in particular that you’d like more information on? What topics?
   4. Where do you go for health information when you have questions or are unsure of something? *Probe – colleagues/ co-workers, internet, reference books?*
      1. Do you ever seek health information online?
         1. If yes, which websites do you most often visit?
   5. Do you know of any resources that pregnant women use to get health information? *Probe – Newspapers? Websites? Women’s groups? Blogs / names of blogs?*

Now I’m going to ask you a few questions about any guidelines or guidance related to testing and treating women for syphilis during pregnancy. As a reminder, there are no right or wrong answers. We are interested in seeing what sorts of guidelines you use in your daily practice, and whether they are useful to you.

- 1. Are there guidelines/ guidance that you follow, related to testing women for syphilis during pregnancy?
     1. If yes, what is the guidance / guidelines?
     2. Who has released those guidelines? *Probe – State/ County health departments? March of Dimes?*
     3. Are there specific guidelines that are helpful to you when you are seeing pregnant women? Which ones? Why?
     4. Are there any guidelines that are not helpful to you? Which ones? Why?
  2. The CDC recently released a phone app with STD treatment guidelines, called “CDC STD TX Guide.”
     1. Have you heard of this app?
     2. Have you downloaded it?
        1. If yes, do you find the app useful? Why / why not?
        2. If no, why did you not download it?

1. **Attitudes about Congenital Syphilis**
2. Among the pregnant women you see, about how many women do you think are at high-risk for having or contracting syphilis while pregnant?
   1. Do you feel that women in your area are at higher risk of syphilis, compared to women throughout the rest of the state? The rest of the United States?
3. When we are talking about a pregnant woman’s risk for having or contracting syphilis, what does the phrase “high-risk” mean to you?
   1. What puts a woman at “high risk” for having or contracting syphilis when pregnant? / When you are seeing a patient, what are the specific things that make you think a woman might be at higher risk for having or contracting syphilis?
4. If you see a woman who is using drugs while pregnant, what are the standard practices that you follow?
   1. Is drug use common among pregnant women in your area?
5. If you see a woman who you suspect may be involved in sex work, what are the standard practices that you follow?
   1. Is sex work common among pregnant women in your area?
6. We know that you are very busy at work and some pregnant women have many competing health priorities. In these cases, how do you decide which health priorities to focus on?
   1. Is there anything that could help you decide which health priorities to focus on? *Probe – Education? Training? Tools? Guidance?*
   2. When you are talking to women with many competing health priorities, what are the things that rank highest in terms of priorities to address?
   3. Where does syphilis generally rank on this priority list?
7. **Practices Related to Syphilis**
8. As part of your job, do you test women for congenital syphilis, or refer them for testing?
   1. If yes, what kinds of tests do you perform?
      1. Are the tests available in house?
      2. Do you refer women to off-site labs for testing?
   2. In which trimester(s) in a woman’s pregnancy do you test?
   3. If a test returns positive, do you treat a woman on the same day that you performed the test?
   4. Are there drugs available for treatment?
      1. If yes, are they free or low cost?
      2. Are they available on-site or are women required to travel to access them?
   5. Have you had any challenges with getting reimbursed for syphilis testing and treatment?
   6. What is your usual procedure/recommendations around partner testing?
   7. Under what circumstances do you **not** test for syphilis?
9. Do you feel that there is a risk of side effects related to treatment for syphilis?
   1. What are the side effects? Why would a woman experience them?
10. Are there any procedures or reporting that you follow if a pregnant woman is found to have syphilis?
11. Do you have to refer women to other hospitals/ clinics for any testing or treatment related to syphilis?
    1. If yes, what types of testing / treatment require referrals to other places?
    2. Where do you refer them?
    3. Are locations typically on-site (at the same location as your hospital/ clinic) or off-site (women are required to travel to complete the testing/ treatment)?
    4. Are there any drawbacks to referring women for testing / treatment?
       1. Do you feel that women typically complete the testing/ treatment when they are referred?
    5. If you order a syphilis test and the patient does not complete the test, are there any follow up procedures to ensure that it gets done? *(Ex: Phone call, letter)*
    6. Do you test all women who deliver a stillborn infant for syphilis?
12. What forms of communication do you mainly use to talk with patients? *Probe: Phone, Email, Hospital messaging system?*
    - 1. What are the most effective ways of reaching patients?
13. Are you aware of any programs or organizations that reach out to pregnant women who may be at risk for contracting STIs?
    1. If yes, which organizations?
    2. Do you think they are effective at engaging this population?
       1. If yes, why? If no, why not?
14. We are interested in finding out the best ways to reach out to pregnant women in Kern County/ East Baton Rouge Parish. Our goal is to make pregnant women more aware of the dangers of congenital syphilis and the rise in cases in Kern County/ East Baton Rouge Parish, and to engage more with prenatal care providers. What do you think is the best way to reach out to pregnant women in Kern County/ East Baton Rouge Parish?
15. Do you have any other suggestions or questions before we end?

**Conclusion:** Thank you so much for your participation! We appreciate the time you took to answer our questions.
